# The C-terminus of *Bienertia sinuspersici* Toc159 contains essential elements for its targeting and anchorage to the chloroplast outer membrane

**DOI:** 10.3389/fpls.2014.00722

**Published:** 2014-12-23

**Authors:** Shiu-Cheung Lung, Matthew D. Smith, J. Kyle Weston, William Gwynne, Nathan Secord, Simon D. X. Chuong

**Affiliations:** ^1^School of Biological Sciences, The University of Hong KongHong Kong SAR, China; ^2^Department of Biology, Wilfrid Laurier UniversityWaterloo, ON, Canada; ^3^Department of Biology, University of WaterlooWaterloo, ON, Canada

**Keywords:** *Bienertia sinuspersici*, Toc159, outer envelope protein, transit peptide, plastid, dimorphic chloroplast, translocon, protein targeting

## Abstract

Most nucleus-encoded chloroplast proteins rely on an N-terminal transit peptide (TP) as a post-translational sorting signal for directing them to the organelle. Although Toc159 is known to be a receptor for specific preprotein TPs at the chloroplast surface, the mechanism for its own targeting and integration into the chloroplast outer membrane is not completely understood. In a previous study, we identified a novel TP-like sorting signal at the C-terminus (CT) of a Toc159 homolog from the single-cell C_4_ species, *Bienertia sinuspersici*. In the current study, we have extended our understanding of the sorting signal using transient expression of fluorescently-tagged fusion proteins of variable-length, and with truncated and swapped versions of the CT. As was shown in the earlier study, the 56 residues of the CT contain crucial sorting information for reversible interaction of the receptor with the chloroplast envelope. Extension of this region to 100 residues in the current study stabilized the interaction via membrane integration, as demonstrated by more prominent plastid-associated signals and resistance of the fusion protein to alkaline extraction. Despite a high degree of sequence similarity, the plastid localization signals of the equivalent CT regions of *Arabidopsis thaliana* Toc159 homologs were not as strong as that of the *B. sinuspersici* counterparts. Together with computational and circular dichroism analyses of the CT domain structures, our data provide insights into the critical elements of the CT for the efficient targeting and anchorage of Toc159 receptors to the dimorphic chloroplasts in the single-cell C_4_ species.

## Introduction

In plant cells, chloroplasts are one of the many types of plastids, which play crucial roles in photosynthesis and other metabolic pathways including amino acid and lipid synthesis, and nitrogen and sulfur assimilation (Keeling, 2004). Therefore, assembly of the correct plastid proteome is crucial for proper functioning of plants and their responses to developmental and external cues. In spite of the presence of a plastid genome, the vast majority of plastid proteins are encoded by the nuclear genome, synthesized in the cytosol as precursor proteins (preproteins), and post-translationally imported into the organelle. The targeting and translocation processes are facilitated by information embedded within the N-terminal sequences of preproteins, known as transit peptides (TPs). In the cytosol, preproteins associate with chaperones (i.e., HSP70 and HSP90; Zhang and Glaser, 2002; Qbadou et al., 2006; Ruprecht et al., 2010) and cochaperones (e.g., HOP and FKBP; Fellerer et al., 2011), and it has been reported that some TPs can be phosphorylated serving as putative binding motifs for 14-3-3 dimers (Waegemann and Soll, 1996; May and Soll, 2000; Martin et al., 2006). At the chloroplast surface, preprotein translocation across the envelope is mediated by the coordinate action of two multiprotein complexes, commonly known as the Translocon at the outer envelope membrane of chloroplasts (Toc) and the Translocon at the inner envelope membrane of chloroplasts (Tic).

The core Toc complex is composed of two GTPases (i.e., Toc159 and Toc34) and a β-barrel protein channel (i.e., Toc75). The two GTPases are also known as the Toc receptors for their cooperative role in controlling the recognition of preproteins, and regulation of preprotein transfer to the translocation channel. TP binding at the GTPase domains (i.e., G-domains) of the Toc GTPases triggers changes in receptor dimerization, GDP/GTP exchange and GTP hydrolysis, ultimately resulting in precursor protein transfer to Toc75 (see Richardson et al., 2014 for review). Despite the homology of the GTPase domains of Toc159 and Toc34, the former has an additional N-terminal acidic domain (i.e., A-domain), which is intrinsically unstructured and highly divergent among isoforms, implicating its ability to distinguish between a wide variety of substrates (Richardson et al., 2009; Dutta et al., 2014). In *Arabidopsis thaliana*, the major (i.e., atToc159) and minor (i.e., atToc90, atToc120, and atToc132) isoforms have been hypothesized to be responsible for the recognition of photosynthetic and housekeeping preproteins, respectively (Ivanova et al., 2004; Kubis et al., 2004; Smith et al., 2004; Infanger et al., 2011). Recently, swapping and yeast-two hybrid studies confirmed that the A-domain of Toc159 is an important determinant of substrate selectivity of the Toc complex (Inoue et al., 2010; Dutta et al., 2014), although, selectivity appears to be conferred by information intrinsic to each preprotein TP, rather than by the function of the protein in chloroplasts (Dutta et al., 2014; Grimmer et al., 2014). On the other hand, the G-domains might constitute a molecular switch as elucidated in many other intracellular protein sorting and translocation processes. The crystal structure of Toc34 G-domain has led to the unraveling of its dimerization properties and functions (Sun et al., 2002; Koenig et al., 2008). While recent biochemical analyses have revealed the relevance of preprotein binding to homodimer dissociation and nucleotide exchange of Toc34 (Oreb et al., 2011), the heterodimeric interaction is crucial for the insertion of Toc159 into the Toc complex with Toc34 serving as a docking site (Bauer et al., 2002; Smith et al., 2002a). In contrast to a single transmembrane α-helix which anchors Toc34 to the chloroplast surface (Kessler et al., 1994; Seedorf et al., 1995), the absence of any hydrophobic cluster raises a question regarding how Toc159 integrates into the chloroplast outer membrane (Bölter et al., 1998; Chen et al., 2000). Conventionally, the entire C-terminal domain (e.g. ~52 kDa in *Pisum sativum*) of the tripartite Toc159 has been referred to as “membrane domain” (i.e., M-domain) solely for its resistance to proteolysis in intact chloroplasts, which implies that it is embedded in a hydrophobic environment (Waegemann et al., 1992; Hirsch et al., 1994; Bauer et al., 2000; Chen et al., 2000). Previously, Lee et al. (2003) demonstrated that the minimal functional unit of Toc159 is constituted by the M-domain, of which overexpression could partially rescue the albino phenotype of the *atToc159* knockout mutant of *A. thaliana* (i.e., *ppi*2). Despite its importance, the study of the M-domain is still in its infancy.

Whilst most of the current knowledge of chloroplast protein import is based on the observations in *P. sativum* and *A. thaliana*, we have recently identified homologs of Toc receptors from the single-cell C_4_ species, *Bienertia sinuspersici* (Lung and Chuong, 2012). This species from the family Chenopodeaceae is of particular interest due to its novel mechanism of C_4_ photosynthesis through subcellular compartmentation of organelles and enzymes within single chlorenchyma cells (Akhani et al., 2005; Chuong et al., 2006). The differential partitioning of nucleus-encoded enzymes between dimorphic chloroplasts implicates the existence of multiple sorting pathways, which could be mediated by the preferential assembly of distinct substrate-specific Toc complexes at distinct subcellular locations (Offermann et al., 2011; Lung et al., 2012). Recently, we showed that the *B. sinuspersici* genome encodes multiple isoforms of Toc159, which are targeted to the dimorphic chloroplasts by a novel C-terminal TP-like sorting signal (Lung and Chuong, 2012). In the current study, we have extended our investigation into the elements of the BsToc159 C-terminus (CT) that are involved in chloroplast targeting and envelope association. We used a number of enhanced green fluorescent protein (EGFP) fusion constructs to differentiate the regions that are required for targeting from those that are important for anchoring the receptor to the chloroplast outer membrane. EGFP fusion proteins with the equivalent regions of the *A. thaliana* homologs and swapping experiments revealed some variation in plastid-associated signals of Toc159 CTs from different species. Overall, our data extend the understanding of the chloroplast targeting information contained within the CT region of Toc159, and reinforce the role it may play in controlling differential subcellular localization to the dimorphic chloroplasts in *B. sinuspersici*.

## Materials and methods

### Plant materials and growth conditions

Seeds from wild-type *A. thaliana* (ecotype Columbia-0) were stratified at 4°C in the dark for 48 h and sowed on 5-cm-tall cell packs containing a 1:1 soil mixture of Sunshine LC1 Mix and Sunshine LG3 Germination Mix (SunGro Horticultural Inc., Bellevue, WA, USA). The plants were maintained in a controlled environment chamber with a day/night photoperiod of 16/8 h at 22°C with a photon flux density of ca. 150 μmol m^−2^ s^−1^and were watered and fertilized regularly with 20:20:20 (N:P:K) fertilizer (Plant Products Co. Ltd., Brampton, ON, Canada). True leaves from 2- to 3-week-old plants were used for protoplast preparation.

### Fluorescent protein fusion constructs

The construction of the AtOEP7-EGFP construct has been described previously (Lung and Chuong, 2012). The other constructs were made by subcloning specific DNA fragments of interest into the pSAT6-35S:DsRed2-N1 or pSAT6-35S:EGFP-C1 vectors (Chung et al., 2005). The transit sequence of ferredoxin was excised from a previous construct (Lung et al., 2011) and subcloned at the 5′ end of the DsRed2-encoding sequence. The C-terminal sequences of Toc159 were obtained by PCR amplification from cDNA clones of the respective isoforms, of which the sequences can be found in the GenBank under the following accession numbers: *B. sinuspersici* Toc159 (JQ739199), *B. sinuspersici* Toc132 (JQ739200), *A. thaliana* Toc159 (AC002330), and *A. thaliana* Toc132 (AC005825). Details of the primers and restriction sites used for generation of the EGFP fusion constructs are listed in Supplementary Table S1. All constructs have been verified by DNA sequencing.

### Biolistic bombardment of onion epidermal cells

Onion (*Allium cepa*) bulbs were purchased from local grocery stores. Briefly, one milligram of tungsten particles (~1.1 μm in diameter; Bio-Rad) were coated with plasmid DNA (EGFP and DsRed2 fusion constructs, 5 μg each) in a suspension containing 16 mM spermidine and 0.1 M CaCl_2_. The DNA-coated tungsten particles were loaded onto the macrocarrier discs and bombarded into the adaxial surface of onion bulb sections (1 cm^2^) from a distance of 12 cm at a pressure of 1350 p.s.i. using a Biolistic PDS-1000/He particle delivery system (Bio-Rad). The bombarded samples were incubated in Petri dishes on moist filter paper at room temperature in the dark for 16 h, and observed under epifluorescence microscopy.

### Protoplast isolation and transfection

The procedures for isolation and transfection of mesophyll protoplasts from *A. thaliana* were modified from Yoo et al. (2007). Briefly, leaves of 3-week-old seedlings were cut into 0.5- to 1-mm strips and incubated in enzyme solution [0.4 M mannitol, 20 mM MES-KOH (pH 5.7), 20 mM KCl, 10 mM CaCl_2_, 0.1% (w/v) bovine serum albumin, 1.5% (w/v) cellulase Onozuka R10 and 0.4% (w/v) macerozyme R10 (Yakult Pharmaceutical, Tokyo, Japan)] at room temperature in the dark for 3 h. The isolated protoplasts were pelleted with equal volume of W5 solution [2 mM MES-KOH (pH 5.7), 154 mM NaCl, 125 mM CaCl_2_ and 5 mM KCl] at 100 g for 2 min, resuspended in W5 solution, and allowed to settle on ice for 30 min. The settled protoplasts were resuspended in MES/Mg^2+^ buffer [0.4 M mannitol, 4 mM MES-KOH (pH 5.7), 15 mM MgCl_2_] at a density of ca. 200,000 protoplasts mL^−1^. Approximately 160,000 protoplasts were mixed with 40 μg of plasmid DNA and 880 μL of polyethylene glycol solution [40% (w/v) PEG4000 (Sigma-Aldrich), 0.4 M sucrose, 0.1 M CaCl_2_]. After incubation at room temperature for 15 min, the transfected protoplasts were mixed with 3.5 mL of W5 solution, pelleted at 100 g for 2 min, resuspended in 4 mL of WI solution [0.5 M mannitol, 4 mM MES-KOH (pH 5.7), 20 mM KCl], and cultured overnight at 23°C with a light intensity of ca. 30 μmol m^−2^ s^−1^. The protoplasts were examined in flat-bottomed depression slides under epifluorescence microscopy.

### Epifluorescence microscopy

Epifluorescence micrographs were acquired using a Zeiss Axio Imager D1 microscope equipped with a Zeiss AxioCam MRm camera (Carl Zeiss Inc., Germany). All images were processed and composed using Adobe Photoshop CS (Adobe Systems Inc.). Representative images were selected from at least three independent experiments. The dual-channel images of transfected onion epidermal cells were analyzed and the corresponding scatterplots, Pearson's correlation coefficients and Manders' coefficients were generated using the open-source Fiji “Colocalization Threshold” plug-in (Schindelin et al., 2012) of Image J software v.1.46 (National Institutes of Health, USA).

### Chloroplast isolation from transfected protoplasts

The procedures for isolating chloroplasts from the transfected protoplasts were modified from Smith et al. (2002b). Briefly, the transfected protoplasts were pelleted with equal volume of W5 solution at 100 g for 2 min, and resuspended in 300 μL of HS buffer [330 mM sorbitol, 50 mM HEPES-KOH (pH 7.3)]. To assemble a protoplast-rupturing device, the needle-fitting end of a 1-mL syringe barrel and the top part of a 500-μL microfuge tube were cut off to form a hollow tube and a slightly wider adaptor ring, respectively. A piece of 10-μm nylon mesh filter (Spectrum Lab Inc.) was fitted against the cut end of the hollow tube and held in place using the adaptor ring. All subsequent steps were carried out at 4°C. The resuspended protoplasts were lysed by passage through the nylon mesh using the protoplast-rupturing device, and the intact chloroplasts were purified on a Percoll step gradient consisting of an upper 500-μL Percoll solution [40% (v/v) Percoll, 50 mM HEPES-KOH (pH 7.3), 330 mM sorbitol, 1 mM MgCl_2_, 1 mM MnCl_2_ and 2 mM EDTA] and a lower 500-μL Percoll solution [85% (v/v) Percoll, 50 mM HEPES-KOH (pH 7.3) and 330 mM sorbitol]. The gradient was centrifuged at 2500 g for 10 min in a swinging-bucket rotor, and the intact chloroplasts at the 40%/85% interface of Percoll were aspirated and diluted with 6 volumes of HS buffer. The isolated chloroplasts were concentrated by centrifugation at 750 g for 5 min and resuspended in 50 μL of HS buffer.

### Subfractionation of isolated chloroplasts into membrane and soluble fractions

The isolated chloroplasts were subfractionated into the membrane and soluble stromal fractions as described previously (Smith et al., 2002b). Briefly, 40 μL of isolated chloroplasts were hypo-osmotically lysed by incubation with 213 μL of 2 mM EDTA on ice for 10 min. To facilitate membrane precipitation, the lysed chloroplasts were mixed with 13.3 μL of 4 M NaCl. After centrifugation at 20,000 g, 4°C for 30 min, the membrane pellet was resuspended in 25 μL of solubilization buffer [50 mM Tris-HCl (pH 8), 5 mM EDTA, 0.2% (w/v) SDS], and the soluble stromal proteins in the supernatant were precipitated with 4 volumes of acetone at −20°C for >1 h and resuspended in 25 μL of solubilization buffer. Similarly, the total protoplast lysates were fractionated into insoluble and soluble fractions using the same procedures.

### Immunoblot analysis

The protein concentrations of all samples were quantified by using Bicinchoninic Acid Protein Assay Kit (Pierce) against standard solutions of bovine serum albumin. The proteins (2.5 μg) were resolved on SDS-PAGE and electroblotted onto polyvinylidene difluoride membranes. The blots were probed with primary antibodies against large-subunit of Rubisco (1:10,000; Agrisera, cat. no. AS03 037), Toc34 (1:16,000; Agrisera, cat. no. AS03 238) or EGFP (1:4000; Lung and Chuong, 2012), followed by a horseradish peroxidase-conjugated anti-rabbit secondary antibody (1:800,000; Sigma-Aldrich, cat. no. 6154). The chemiluminescence signals were produced using Amersham ECL-Advance solution (GE Healthcare) and captured by exposing the blots to Amersham Hyperfilm ECL films (GE Healthcare), which were developed using a CP1000 Agfa photodeveloper (AGFA). The films were scanned and processed using Adobe Photoshop CS (Adobe Systems). The intensities of immunoreactive bands were densitometrically quantified using the gel-analyzer function of ImageJ software v.1.46 (National Institutes of Health, USA).

### Expression and purification of recombinant AtToc159M_His_

The M-domain of Toc159 was obtained by PCR amplification from the Arabidopsis cDNA (AC002330) and subcloned into the pET28a(+) expression vector (Novagen) for production of hexahistadine-tagged recombinant protein (AtToc159M_His_). The recombinant protein was purified by immobilized metal ion affinity chromatography (IMAC) under denaturing conditions using the Profinity™ IMAC Ni^2+^-charged resins (Bio-Rad). The purified sample of AtToc159M_His_ was dialyzed against CD buffer [10 mM Tris-HCl (pH 7.5), 5 mM MgCl_2_, 50 mM NaCl, 1 mM DTT].

### Circular dichroism

Circular dichroism spectra were recorded for the recombinant AtToc159M_His_ protein in the range 190–260 nm using an Aviv 215 spectrometer (Aviv Associates Inc.) and a quartz cuvette of 0.005 cm path length. Two independent samples of AtToc159M_His_ at 9 μM were tested; 4 scans at 0.5 nm/s were made at 0.5 nm intervals for each sample. And the spectra were averaged. The percentage of secondary structure was calculated by deconvoluting the averaged circular dichroism spectra using the online DICRHOWEB CD secondary structure server (Whitmore and Wallace, 2004).

## Results

### The CT of Toc159 contains chloroplast-targeting and chloroplast membrane-associating information

**Figure 1:**
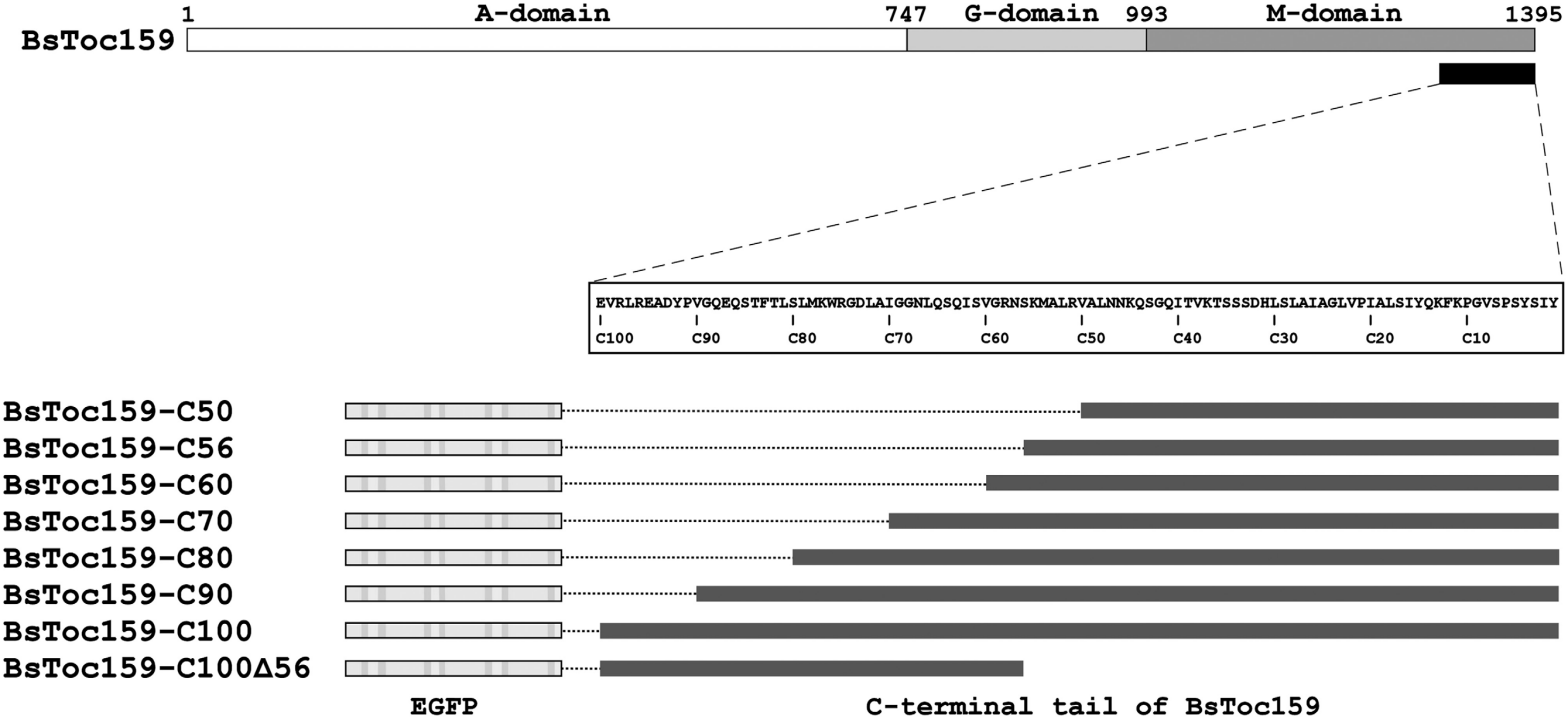
**Schematic overview of EGFP fusion constructs with various lengths and regions of BsToc159 C-terminus**. Toc159 exhibits a tripartite structure consisting of an N-terminal acidic (A) domain, a central GTPase (G) domain and a C-terminal membrane-anchor (M) domain. Various lengths of the BsToc159 C-terminus from 50 to 100 residues (i.e., C50 to C100) and a truncated region (i.e., C100Δ56) were fused to the C-terminus of EGFP in the pSAT6 vector for 35S promoter-driven expression.

**Figure 2:**
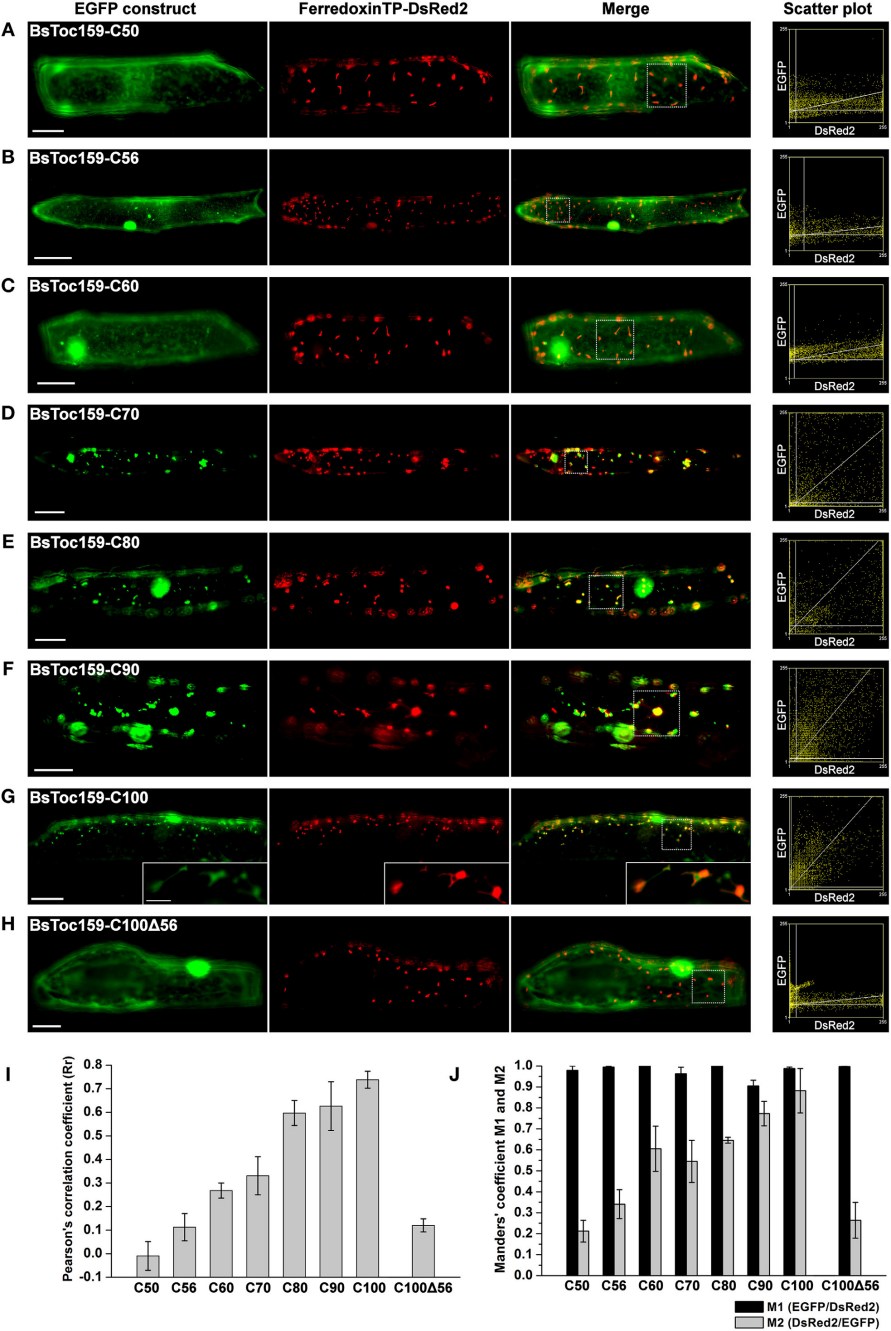
**Colocalization analysis of EGFP fusion proteins with BsToc159 C-terminal regions in onion epidermal cells**. Onion bulb scales were biolistically co-transformed with an EGFP fusion construct containing the **(A)** BsToc159-C50, **(B)** BsToc159-C56, **(C)** BsToc159-C60, **(D)** BsToc159-C70, **(E)** BsToc159-C80, **(F)** BsToc159-C90, **(G)** BsToc159-C100, or **(H)** BsToc159-C100Δ56 and a DsRed2 fusion construct with the ferredoxin transit peptide for transient protein expression driven by the constitutive 35S promoter. For each construct, representative images of EGFP (green), DsRed2 (red) and a merge of the two channels are shown. Colocalization of the green and red signals produced yellow signals. Scatter plots show the distribution of the green and red pixels in the sample areas of the merge panels as indicated in dotted line boxes. The x- and y-axes indicate the intensities of the red and green pixels, respectively, on the range of pixel gray values from 0 to 255. The clustering of pixels from both channels along a diagonal line represents colocalization. **(I)** The Pearson's correlation coefficients (R_*r*_) of the two fluorescent channels. The maximum theoretical R_*r*_ score is 1. The values represent the mean of four replicates (± SE). **(J)** Manders' coefficients M1 and M2. M1 indicates the fraction of green pixels which colocalized with red pixels, and M2 indicates the fraction of red pixels which colocalized with green pixels. The values represent the mean of four replicates (± SE). Scale bars = 50 and 5 μm (inset).

Our previous bioinformatics analyses predicted that the CT of BsToc159 shares similar physicochemical and structural properties with chloroplast TPs (Lung and Chuong, 2012). Specifically, a putative TP-like chloroplast-sorting signal of 51 amino acids together with a putative stromal processing peptidase cleavage site was identified using the neural network-based ChloroP predictor (Emanuelsson et al., 1999). Accordingly, our previous EGFP-fusion experiments were based on the ChloroP-predicted TP-like region at the CT of BsToc159 plus five additional residues, which successfully directed the reversible association of the passenger proteins with the outer envelope of chloroplasts (Lung and Chuong, 2012). It is a common practice to include some residues from the mature protein downstream of the predicted TP cleavage site when studying targeting of typical chloroplast preproteins (e.g., Lee et al., 2003, 2006, 2009). While the exact number of residues that should be included is not known, the original decision to include 5 additional residues beyond the predicted 51-amino acid TP-like chloroplast sorting signal of BsToc159 (Lung and Chuong, 2012) was based on other studies where approximately 5 amino acids were included (e.g., Ivanova et al., 2004; Smith et al., 2004; Inoue et al., 2010; Okawa et al., 2014). In the present study, we sought to further elucidate the functional region of the novel TP-like sorting signal used by BsToc159 and identify the essential region which mediates the successful integration of the receptor into the outer envelope membrane of chloroplasts. First, we produced a number of transient expression constructs by fusing various lengths of BsToc159 CT ranging from 50 to 100 residues (i.e., C50 to C100) to the CT of EGFP (Figure 1). To evaluate the efficiency of the variable lengths of BsToc159 CT as a plastid-sorting signal, the EGFP-fusion constructs were subjected to colocalization studies in onion epidermal cells, which were co-transformed with a DsRed2 construct fused with the ferredoxin TP to direct it to plastids (Figure 2). Interestingly, although we previously showed that the 56 most C-terminal residues (i.e., the C56 construct) could direct ca. 60% of EGFP signal to the chloroplast envelope using *A. thaliana* mesophyll protoplasts (Lung and Chuong, 2012), the diffuse fluorescent signals of C50, C56 and C60 fusion proteins indicated a cytoplasmic localization in onion epidermal cells (Figures 2A–C). Thus, the contrasting subcellular localization patterns of the C56 construct in the two cell types implicated some species-specific preferential targeting of the BsToc159 CT. When the BsToc159 CT was extended to include additional upstream residues (i.e., C70, C80 and C90), the fusion proteins appeared as punctate fluorescent spots, some of which were colocalized with the DsRed2-decorated plastids (Figures 2D–F). The non-plastid punctate structures that did not colocalize with the DsRed signals appeared irregular in size and shape most likely representing insoluble aggregates due to protein misfolding (Figures 2D–F). However, a general trend was apparent in that the proportion of plastid-localized EGFP signal increased with the length of the BsToc159 CT fusion from C70 to C90 (Figures 2D–F). As the length increased to C100, the vast majority of the EGFP signals colocalized with the DsRed2-decorated plastids as is evident from the merge of the two channels producing yellow punctate signals and the diagonal scattering pattern of pixels from both channels in a scatter plot (Figure 2D). Occasionally, the C100 fusion proteins also labeled tubular protrusions extending from the DsRed2-decorated plastids, reminiscent of stroma-filled extensions called stromules (Figure 2G inset; Köhler and Hanson, 2000). On the other hand, deletion of the C-terminal 56 residues of BsToc159 from the C100 construct completely abolished plastid-targeting, leading to diffuse EGFP signals, further confirming that this region contains key plastid-targeting information (Figure 2H). Quantitatively, the Pearson's correlation coefficients and the Manders' coefficients (Manders et al., 1993) confirmed that the C100 fusion protein was among the best colocalized with the plastids (Figures 2I,J).

**Figure 3:**
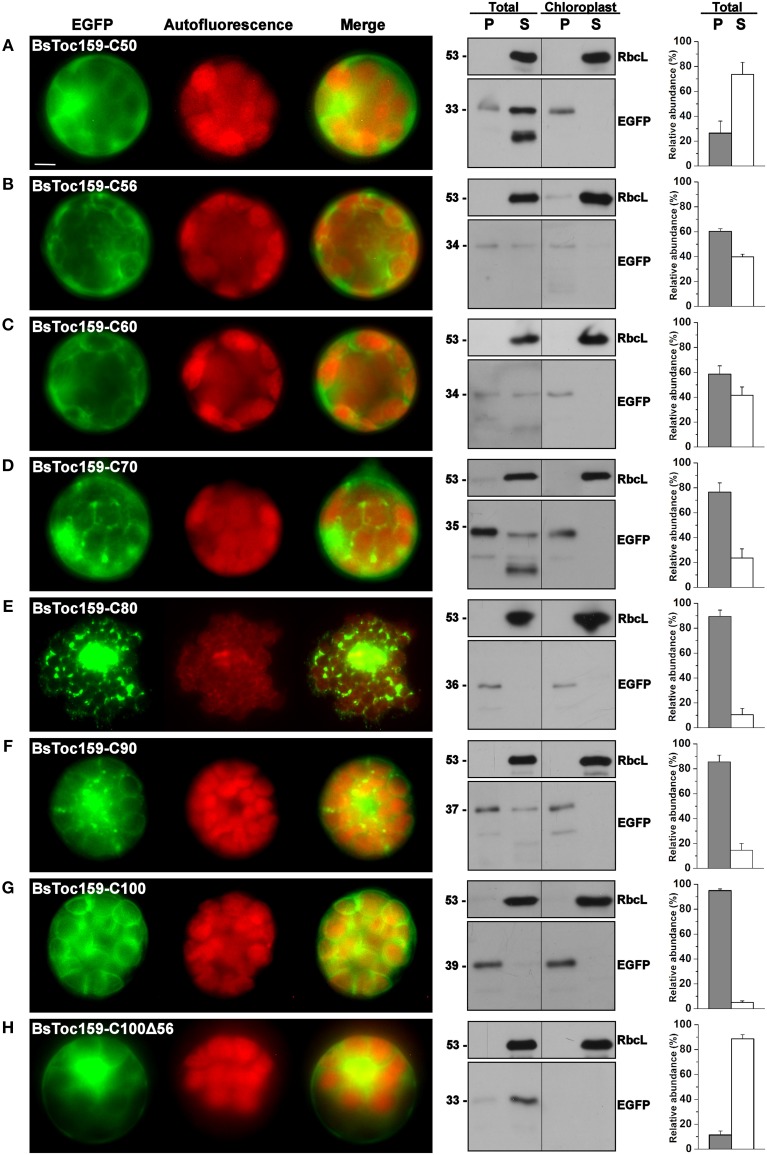
**Transient expression of EGFP fusion proteins with BsToc159 C-terminal regions in *A. thaliana* protoplasts**. Isolated protoplasts were transfected with various EGFP fusion constructs containing the **(A)** BsToc159-C50, **(B)** BsToc159-C56, **(C)** BsToc159-C60, **(D)** BsToc159-C70, **(E)** BsToc159-C80, **(F)** BsToc159-C90, **(G)** BsToc159-C100, or **(H)** BsToc159-C100Δ56 for transient protein expression driven by the constitutive 35S promoter. For each construct, representative images of EGFP (green) and chlorophyll fluorescence (red) and a merge of the two channels are shown in the left panel. The subcellular localization was confirmed by immunoblot analysis with an anti-EGFP antibody after subfractionation of the total protoplasts or purified chloroplasts in pellet (P) and soluble (S) fractions (middle panels). Detection with an antibody against Rubisco large subunit (RbcL) served as loading controls for the soluble fractions. The immunoreactive bands of the total protoplast subfractions were densitometrically quantified (right panels). Each value represents the mean of three replicates (± SE). Scale bar = 10 μm.

**Figure 4:**
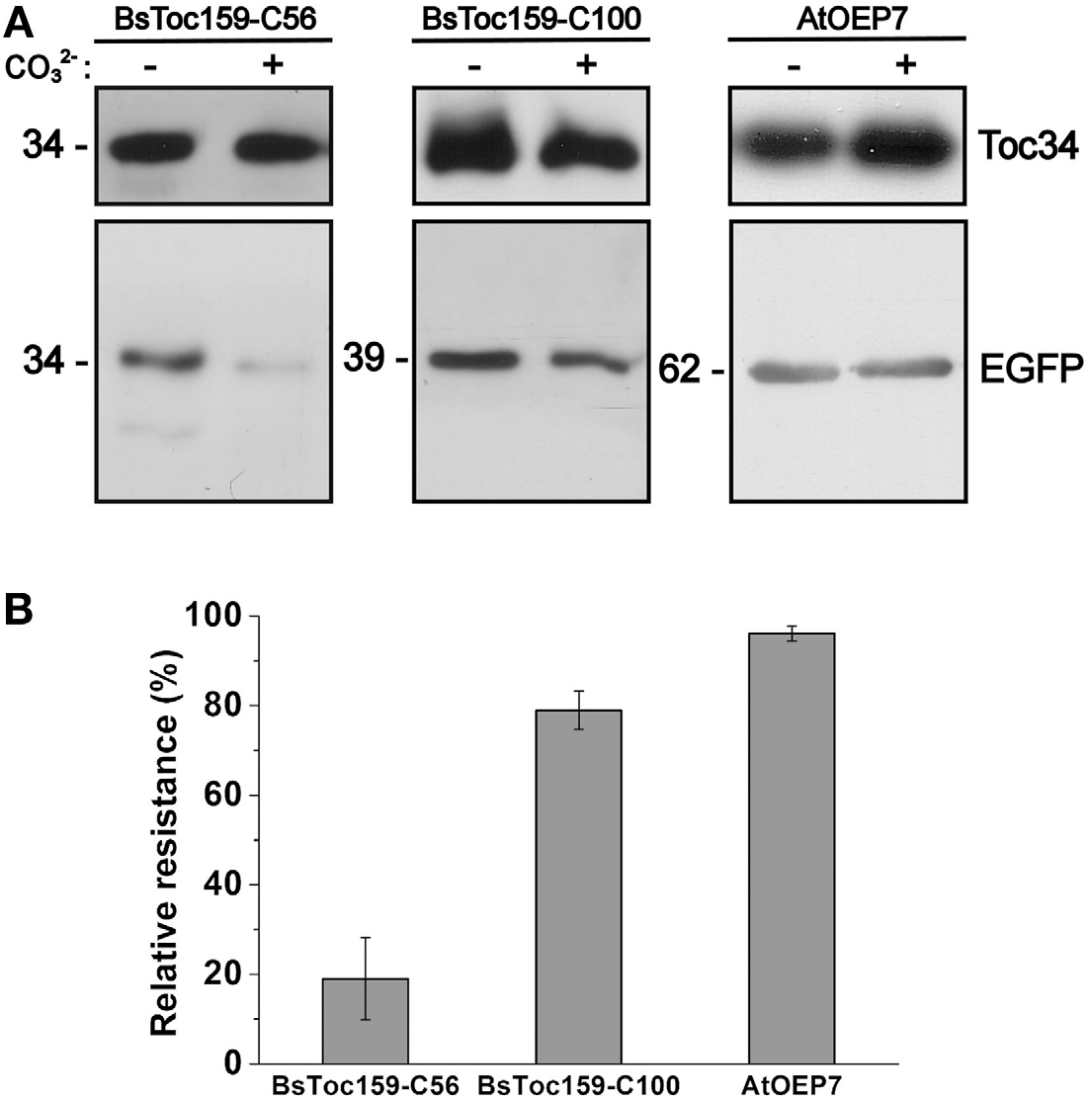
**Alkaline extraction of chloroplasts purified from transfected *A. thaliana* protoplasts. (A)** Chloroplasts were isolated from transfected protoplasts that expressed the C-terminal 56- or 100-residues of BsToc159 fused to the C-terminus of EGFP (left and middle panels), or AtOEP7, an integral outer envelope protein, fused to the N-terminus of EGFP (right panel). Purified chloroplasts were incubated in CO^2−^_3_ buffer (pH 11.5) or HEPES buffer (pH 7.3) on ice for 10 min. The precipitated membrane pellets were subjected to immunoblot analysis using an anti-EGFP antibody and an antibody against Toc34 as loading controls. **(B)** The relative resistance of the three EGFP fusion constructs to alkaline extraction as determined by densitometric analysis of the immunoreactive bands. Each value represents the mean of three replicates (± SE).

To evaluate the chloroplast-targeting efficiency of the different regions of the BsToc159 CT, the same EGFP-fusion constructs were transiently expressed in *A. thaliana* mesophyll protoplasts. The transfected protoplasts were observed using fluorescence microscopy and the EGFP signals were also densitometrically measured following immunoblot analysis of transfected protoplasts fractionated into soluble and insoluble fractions (Figure 3). Under the microscope, the C50 fusion proteins were predominantly observed as diffuse signals with approximately a quarter of the signal detected in the chloroplast membrane-associated fraction (Figure 3A). Increasing the length of the BsToc159 CT by 6 residues (i.e., C56) effectively directed 60% of EGFP protein to the chloroplast surface, resulting in the ring-like appearance of fluorescent signals surrounding the chloroplasts (Figure 3B). Further increase of the BsToc159 CT by 4 residues (i.e., C60) did not alter the subcellular distribution of EGFP signals qualitatively or quantitatively as compared to the C56 construct, suggesting that the required targeting information of the CT is contained within the C-terminal 56 residues of BsToc159 (Figure 3C). The presence of a strong signal in the soluble fraction for the C56 and C60 constructs could be attributed to the absence of a chloroplast membrane anchor, rendering their association with the chloroplast envelope transient/reversible. Alternatively, the elevated levels of these constructs in the soluble fraction could be due to overexpression and therefore slow targeting of the proteins to the chloroplasts leading to cytosolic accumulation. This observation is in agreement with our previous findings that the envelope-associated C56 fusion proteins were susceptible to alkaline extraction, and that the addition of the Toc34 G-domain to the fusion protein effectively boosted the chloroplast membrane-associated signals to over 80% (Lung and Chuong, 2012). Similar to our observations in onion epidermal cells, the C70, C80 and C90 constructs produced irregular punctate aggregates in addition to the ring-like signals encircling the chloroplasts (Figures 3D–F). Among the three constructs, the fluorescent signals at the chloroplast exterior were most prominent with C90 (Figure 3F). The fluorescent signals of the C100 construct were exclusively localized to the chloroplast envelope, whereas removal of the predicted chloroplast-targeting signal (i.e., C100Δ56) from this construct completely abolished chloroplast targeting, as expected (Figures 3G,H). The significantly higher abundance of chloroplast membrane-associated C100 signals compared to that of the C56 could be attributed to the presence of a membrane-anchoring region stabilizing the association between the C100 fusion proteins and the chloroplast envelope. In fact, alkaline extraction of the chloroplasts isolated from transfected protoplasts prior to immunoblot analysis revealed a drastic difference in the relative resistance of the C56 (i.e., 20%) as compared to the C100 (i.e., 80%) fusion protein (Figure 4). Taken together, we believe that the essential and sufficient chloroplast-sorting information for BsToc159 is embedded within the C-terminal 56 residues, whereas the immediate upstream sequence is important for anchoring the protein to the chloroplast surface, potentially by an as yet undetermined membrane-associating structure(s), which may not be complete or folded properly in the truncation constructs C70, C80 and C90, leading to the formation of insoluble aggregates (Figures 2D–F, 3D–F).

### The M-domain of Toc159 forms an unconventional membrane-anchor

**Figure 5:**
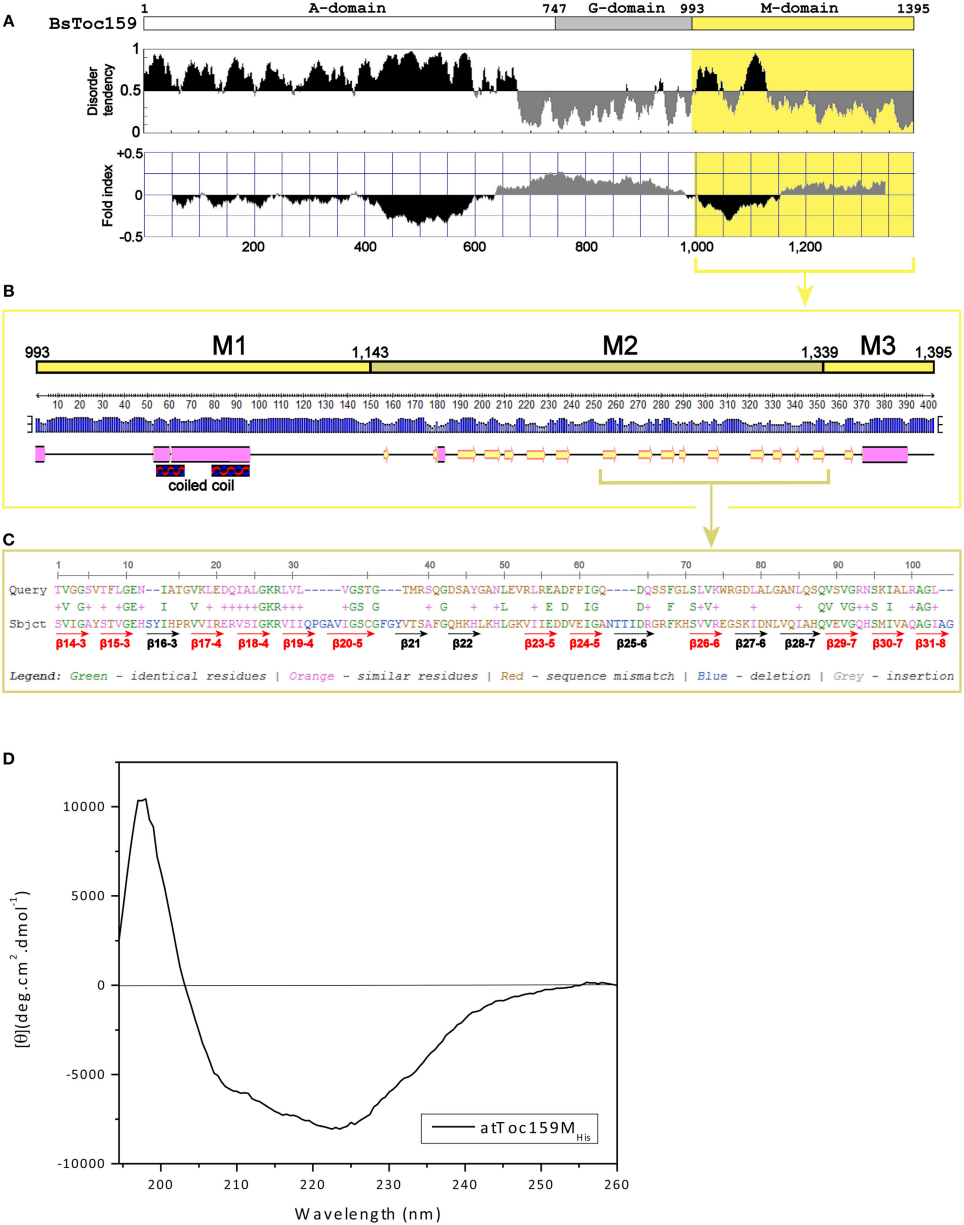
**Prediction of the M-domain structure. (A)** Prediction of structurally disordered regions of BsToc159. IUPred (Dosztanyi et al., 2005; upper panel) and FoldIndex (Prilusky et al., 2005; lower panel) were used to predict the intrinsically disordered (shaded in black) and structured (shaded in gray) regions of the entire BsToc159 protein. The M-domain region is shaded in yellow. **(B)** Secondary structure prediction of the BsToc159 M-domain. Predictions were performed using the PSIPRED protein structure prediction server v3.0 (Jones, 1999). The height of the blue bar for each residue represents the confidence level. Cylinders, arrows and lines symbolize α-helices, β-strands and coils, respectively. Based on the structural prediction, the M-domain is subdivided into the M1, M2 and M3 subdomains: M1 represents a moderately disordered region with a putative α-helical region, which was predicted to fold into a coiled-coil structure by COILS (Lupas et al., 1991); M2 represents a β-strand-rich region; M3 represents the C-terminal 56-residue sorting signal. **(C)** Amino acid sequence alignment of M2 region with the lipid-binding domain of UDP-3-*O*-acyl-glucosamine *N*-acyltransferase (LpxD). BLAST search was performed using the AtToc159 M-domain as query sequence against 3D structures deposited in the RCSB Protein Data Bank (http://www.pdb.org/pdb/search/searchSequence.do). The β-strands which constitute the left-handed β-helix of LpxD (Buetow et al., 2007) are annotated by red (if homologous to M2) and black arrows (otherwise). **(D**) Far-UV CD spectrum of purified recombinant AtToc159M_His_. The protein concentration was 9 μM. MRE is the mean residue ellipticity in degrees cm^2^ dmol^−1^. Temperature was 25°C.

To complement our findings from the truncation experiments, we further investigated the structure of the Toc159 M-domain (Figure 5). While the A-domain has been characterized as an intrinsically unstructured domain (Richardson et al., 2009) and the G-domain structure can be deduced from the crystal structure of its GTPase homolog Toc34 (Sun et al., 2002; Reddick et al., 2007; Yeh et al., 2007; Koenig et al., 2008), the structure of the M-domain has not been studied previously. First, to gain insight into its structural organization, the amino acid sequence of BsToc159 was analyzed using IUPRed (Dosztanyi et al., 2005) and FoldIndex (Prilusky et al., 2005) to predict intrinsically disordered and structured regions (Figure 5A). Concomitantly with the use of a neural network predictor for protein secondary structures by the PSIPRED algorithm, we further divided the M-domain of BsToc159 into three subdomains designated as M1, M2 and M3 (Figure 5B). The N-terminal region of the M-domain is linked to the central G-domain via a 150-residue M1 region, which is moderately unstructured except for a putative α-helical motif arranged in a predicted coiled-coil structure (Figures 5A,B), whereas the C-terminal 56-residue region containing the chloroplast targeting signal, designated as M3, contains a predicted amphipathic α-helix, which is also a structural feature of TPs (Figures 5A,B; Lung and Chuong, 2012). The core region of the M-domain, designated as the M2 subdomain, is predicted to be a β-strand-rich region (Figure 5B). Since the resistance of the C100 fusion protein to alkaline extraction also implied that some structural features within part of this subdomain might be involved in chloroplast outer membrane association (Figure 4), we asked if the M2 subdomain had a tendency to fold into a β-barrel, which is a common conformation comprised of multiple amphipathic β-strands that span the outer membranes of gram-negative bacteria and endosymbiotic organelles (Walther et al., 2009). However, the M-domains of BsToc159 and *A. thaliana* homologs have a negligible probability (*P* = 0.05) of adopting a transmembrane β-barrel conformation, according to the PROFtmb prediction program (http://www.predictprotein.org; Bigelow et al., 2004). On the other hand, a BLAST search of the structural database deposited in the Protein Data Bank (PDB) using the *A. thaliana* Toc159 M-domain as query sequence revealed considerable homology (i.e., 47%) of the M2 subdomain with the lipid-binding domain of UDP-3-*O*-acyl-glucosamine *N*-acyltransferase (LpxD), which is predominantly composed of β-strands (Figure 5C; Buetow et al., 2007), and is consistent with our prediction that M2 is a β-strand-rich region (Figure 5B). Interestingly, LpxD belongs to a rare family of left-handed β-helical proteins in which each coil is formed by three hexapeptide repeats of a consensus sequence (Buetow et al., 2007).

The secondary structure of purified recombinant AtToc159M refolded in 1% LDAO was then examined using far-UV CD spectroscopy. Qualitatively, the spectrum is indicative of the presence of α-helical elements, depicted by the double minima ellipticity at 208 nm and 222 nm, as well as β-strand elements, based on the minimum ellipticity at approximately 215 nm (Figure 5D; Park et al., 1992). Overall, the spectrum indicates an ordered, but complex, conformation comprised of a combination of helical, sheet and turn elements. The relatively lower minimum ellipticity at 208 nm, in comparison with the minimum at 222 nm, could indicate a tight interaction between the secondary structures and/or the possibility of self-association of the protein (Hoang et al., 2012, 2013). Deconvolution of the spectra using two different reference sets of known CD spectra suggests that AtToc159M is comprised of approximately 39.5% α-helical, 20% β-strand, 21% unfolded and 17.5% turn elements (Supplementary Table S2). This is in agreement with sequence-based structure (PSIPred) and disorder predictions implicating an α-helical segment within subdomain M1, a smaller segment in the middle of M2, and another large predicted α-helix near the CT of M3 (Figure 5B). While these predicted α-helical domains do not appear to amount to 40% of the entire M-domain, the N-terminal helical segment is predicted to form a coiled-coil structure. The qualitative shape of the curve is similar to that of previously characterized coiled-coil proteins, and it is possible that such a structure accounts for the high apparent α-helical content suggested by the deconvolution (Greenfield and Hitchcock-DeGregori, 1993). The prediction that the M-domain contains significant regions of disorder (i.e., segments flanking the coiled-coil region of M1), and β-strand (i.e., large proportion of the M2 subdomain) is also supported by the deconvolution of the CD spectrum (Figures 5A,B). Taken together, these findings led us to hypothesize that an independent region upstream of the CT TP-like sorting signal adopts a conformation that is involved in the interaction of Toc159 with the chloroplast outer membrane.

### The CT of BsToc159 displays species-specific targeting

**Figure 6:**
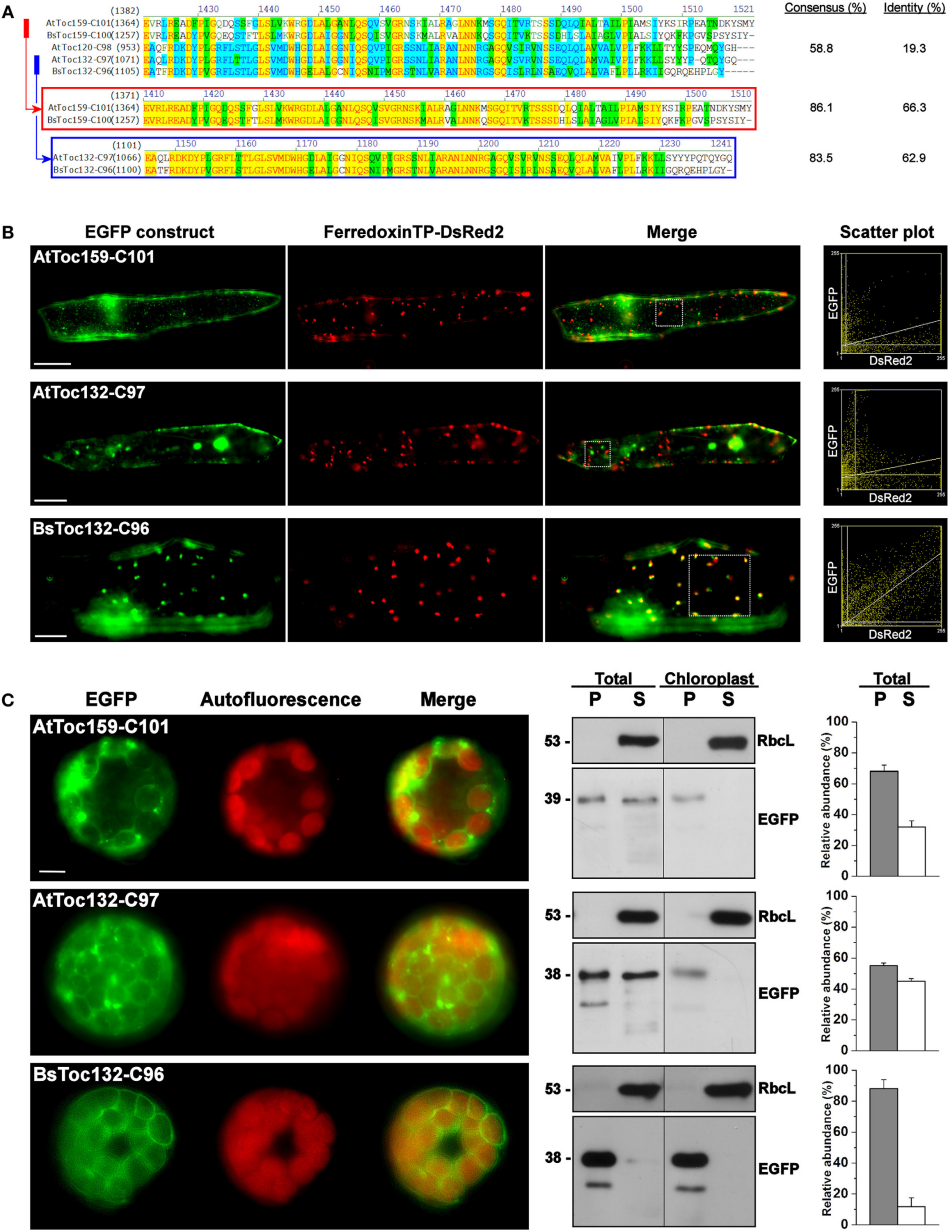
**Transient expression of EGFP fusion proteins with the C-terminal regions of other Toc159 homologs. (A)** Amino acid sequence alignment of the Toc159 homologs from *B. sinuspersici* and *A. thaliana*. Sequence homologies among the Toc159 and Toc132 isoforms are shown in red and blue boxes, respectively. Alignment was performed using the AlignX module of Vector NTI Advance™ 10.3.0 (Invitrogen) and is displayed using the default color scheme: a red foreground on a yellow background denotes a 100% conserved residue; a dark green foreground on a white background denotes a residue with weak similarity to the consensus residue at a given position; a black foreground on a light green background denotes a consensus residue in a block of similar residues at a given position; A blue foreground on a cyan background denotes a conserved residue with 50% or higher identity at a given position; A black foreground on a white background denotes a non-similar residue. **(B)** Colocalization analysis of EGFP fusion proteins in onion epidermal cells. EGFP was fused to the C-terminal regions of other Toc159 homologs equivalent to the EGFP-BsToc159-C100 construct based on the protein alignment as shown in **(A)**. Details are the same as in Figure 2. **(C)** Transient expression of EGFP fusion proteins in isolated *A. thaliana* protoplasts. Details are the same as in Figure 3.

Consequently, we further investigated if the membrane-association motif and the TP-like sorting signal identified within the C-terminal 100 residues of BsToc159 are present in other Toc159 homologs. Amino acid sequence alignment of multiple Toc159 homologs from *B. sinuspersici* and *A. thaliana* illustrated high homology of the C-terminal regions among members of the same Toc159 subtype (Figure 6A). For instance, the C-terminal 100 residues of BsToc159 exhibit 86% similarity with the aligned region of AtToc159, whereas the equivalent regions of BsToc132 and AtToc132 share 83.5% similarity (Figure 6A). In both cases, pairwise comparison revealed that sequence variation is primarily found at the CT ends (Figure 6A). On the other hand, the primary sequences are more divergent when comparing the two subtypes (i.e., Toc159 vs. Toc132) which share 58.8% overall similarity and only 19.3% identity (Figure 6A). From the sequence alignment, we defined the equivalent regions corresponding to BsToc159-C100 from the other *A. thaliana* (i.e., AtToc159-C101 and AtToc132-C97) and *B. sinuspersici* (i.e., BsToc159-C100 and BsToc132-C96) homologs for EGFP fusion studies in onion epidermal cells (Figure 6B) and Arabidopsis mesophyll protoplasts (Figure 6C). The fluorescence micrographs showed that the subcellular localization patterns of BsToc132-C96 (Figures 6B,C) were qualitatively and quantitatively indifferent from that of BsToc159-C100 (Figures 2G, 3G), with strong association of the fusion proteins with the etioplasts of onion epidermal cells and the chloroplast envelopes of mesophyll protoplasts. Surprisingly, AtToc159-C101 and AtToc132-C97 did not produce strong plastid-associated signals in spite of their high primary sequence consensus with BsToc159-C100 and BsToc132-C96, respectively (Figures 6B,C). Neither the AtToc159-C101 fusion protein nor the AtToc132-C97 equivalent colocalized with the DsRed2-decorated etioplasts in onion epidermal cells (Figure 6B). In mesophyll protoplasts transfected with AtToc159-C101 or AtToc132-C97 constructs, although fluorescent signals were observable surrounding the chloroplasts, considerable signals were also detected in the soluble fractions as well as associated with non-plastid punctate structures (Figure 6C). Taken together, we conclude that the CTs of both *B. sinuspersici* Toc159 homologs (i.e., BsToc159 and BsToc132) could mediate the targeting and stable association of EGFP fusion proteins with etioplasts and chloroplast envelopes, whilst the homologous counterparts from *A. thaliana* produced less conclusive results.

**Figure 7:**
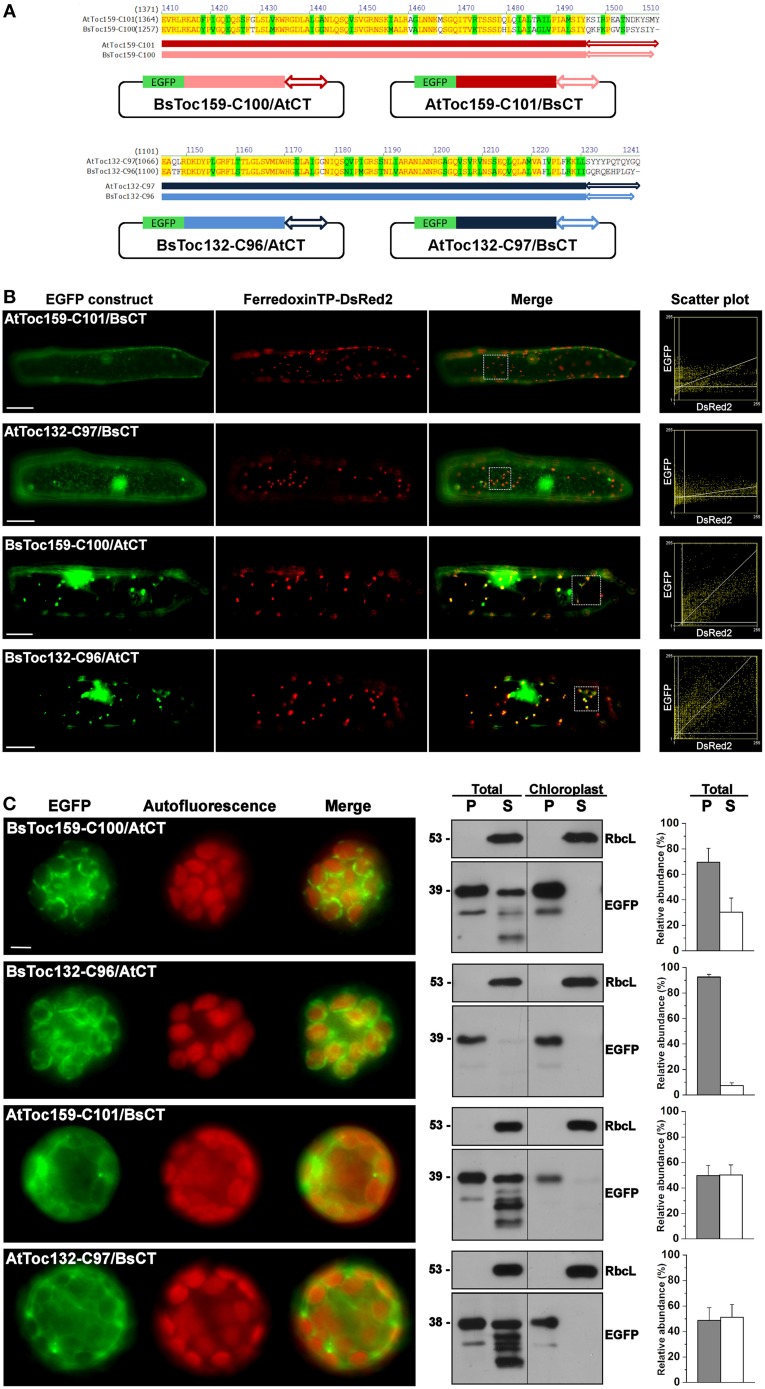
**Swapping of the short C-terminal tails between Toc159 homologs. (A)** Schematic representation of the C-terminal swapping constructs. Based on the amino acid sequence alignment (see details in Figure 6A), the highly variable C-terminal ends, as depicted by double arrows, were swapped between the *B. sinuspersici* and *A. thaliana* isoforms. The Toc159 and Toc132 isoforms are depicted in red and blue, respectively. **(B)** Colocalization analysis of EGFP fusion proteins in onion epidermal cells. Details are the same as in Figure 2. **(C)** Transient expression of EGFP fusion proteins in isolated *A. thaliana* protoplasts. Details are the same as in Figure 3.

Due to the dissimilar plastid targeting results when using the CTs of Toc159 homologs from different species, we finally asked if the highly divergent 10- to 15-residues at the end of the CT tails constitute an important part of the plastid-targeting signal. Chimeric constructs were made by swapping the CT tails of BsToc159-C100 and AtToc159-C101, as well as between BsToc132-C96 and AtToc132-C97 (Figure 7A). In onion epidermal cells, both BsToc159-C100 and BsToc132-C96 efficiently directed EGFP to the etioplasts regardless of the swapped CT tails from *A. thaliana* homologs, whilst AtToc159-C101 and AtToc132-C97 could not guide EGFP to plastids despite the presence of *B. sinuspersici* CTs (Figure 7B). In mesophyll protoplasts, the targeting of BsToc159-C100 to the chloroplast outer membrane was only slightly diminished by swapping the CT domain with that of AtToc159 (compare Figure 7C with Figure 3G). In addition, replacing the CT domain of BsToc132 with that of AtToc132 also did not produce any observable effect on chloroplast targeting of BsToc132-C96 (compare Figure 7C with Figure 6C). On the other hand, replacing the CT of AtToc159-C101 and AtToc132-C97 with those of the corresponding *B. sinuspersici* CTs did not improve chloroplast targeting of the Arabidopsis proteins (compare Figure 6C with Figure 7C). The stronger plastid-associated signals obtained using the *B. sinuspersici* constructs compared to those of *A. thaliana* might be attributed to the species-specific sequence differences within the upstream region which stabilize chloroplast envelope association, independently of the highly divergent CT sequence which constitutes the sorting information. In fact, our previous data confirmed that the CTs of AtToc159 and AtToc132 could effectively re-target a Toc34 mutant protein to the chloroplast envelope, suggesting the presence of sufficient chloroplast-sorting information within their sequences (Lung and Chuong, 2012). In *B. sinuspersici*, the more stable chloroplast envelope association as mediated by the putative single-site variants could have some implications on the insertion of Toc159 receptors into the outer membrane of the dimorphic chloroplasts for differential preprotein targeting.

## Discussion

### Toc159 CT represents a new class of sorting signal to the chloroplast outer membrane

Our recent discovery of the chloroplast-targeting information embedded within the BsToc159 CT using sequence-based bioinformatics predictions (Lung and Chuong, 2012) raises a number of fundamental questions about the nature of this novel sorting signal. For instance, what is the length of the signal that is essential for chloroplast sorting? Is there a membrane-integration element associated with the signal that is responsible for anchoring the receptor to the chloroplast envelope? Is this signal unique to the Toc159 isoform of the single-cell C_4_ species? In the current study, we have addressed these questions using transiently-expressed fluorescent proteins fused to variable-length truncation and domain-swapped constructs, and have complemented this approach with structural analyses of the M-domain. Collectively, our data point to a novel class of sorting signals present in the Toc159 family of chloroplast protein import receptors for targeting to the chloroplast outer membrane. According to the Plant Proteome Database, approximately 47 different proteins are annotated to reside on the chloroplast outer membrane (http://www.plantsciences.ucdavis.edu/kinoue/OM.htm; http://ppdb.tc.cornell.edu; Inoue, 2007; Sun et al., 2009; Breuers et al., 2011; Inoue, 2011). Although the mechanisms for targeting of these outer envelope proteins (OEPs) have not been completely elucidated, multiple pathways are apparent and, in many cases, the membrane-spanning domains constitute the protein sorting information (for reviews, see Hofmann and Theg, 2005; Bölter and Soll, 2011; Lee et al., 2013). With the exception of Toc75, which relies on an N-terminal TP for chloroplast targeting (Tranel et al., 1995), the other identified integral β-barrel proteins, including OEP21, OEP24 and OEP37, appear to self-insert into the chloroplast outer membrane (Pohlmeyer et al., 1998; Bölter et al., 1999; Goetze et al., 2006). The majority of α-helical OEPs commonly contain a single hydrophobic α-helix which functions as a transmembrane anchor as well as a sorting signal (Hofmann and Theg, 2005; Bölter and Soll, 2011). Depending on whether the transmembrane domain is located at the N- or CT, these OEPs are broadly classified into the families of signal-anchored proteins (e.g., OEP7, OEP14, HKI, Toc64, CHUP1) (Li et al., 1991; Wiese et al., 1999; Sohrt and Soll, 2000; Lee et al., 2001; Oikawa et al., 2008) and tail-anchored proteins (e.g., OMP24, HPL, Toc34, OEP9) (Fischer et al., 1994; Chen and Schnell, 1997; Froehlich et al., 2001; Dhanoa et al., 2010), respectively. Although it had been originally proposed that these proteins are spontaneously integrated into the destination membrane without any energy requirement or proteinaceous factor (Schleiff and Klösgen, 2001), Bae et al. (2008) discovered a chaperone-like ankyrin repeat protein (i.e., AKR2A) in *Arabidopsis* which binds to the transmembrane domains and the CT regions of tail-anchored proteins, and thereby functions as a cytosolic mediator for their specific sorting to the chloroplast envelope. To meet the criteria of tail-anchored proteins, a protein must exhibit three structural features: (i) the exposure of the majority of the protein to the cytosolic side; (ii) the presence of a single transmembrane domain at or near the CT, and; (iii) the protrusion of a short CT tail into the organelle interior (Kutay et al., 1993; Abell and Mullen, 2011). Despite the fact that Toc159 shares some structural resemblance to a tail-anchored protein and its GTPase homolog, Toc34, is a tail-anchored protein (Dhanoa et al., 2010), our studies revealed some important differences. First, we showed that the BsToc159 CT contains some chloroplast-sorting information, but this region does not appear to constitute a hydrophobic transmembrane α-helix (Lung and Chuong, 2012), as demonstrated by the susceptibility of BsToc159-C56 fusion proteins to alkaline extraction (Figure 4). In this study, we identified a discrete region within the 60–100 residues from the CT of BsToc159 that constitutes a membrane association domain, as demonstrated by the resistance of BsToc159-C100 proteins to alkaline extraction (Figure 4). Contrary to that of tail-anchored proteins, this membrane-associating region does not contain an insertion signal for the outer membrane of the plastid envelope since the C-terminally truncated construct (i.e., BsToc159-C100Δ56) produced cytosolic localization of the EGFP proteins (Figures 2H, 3H). Previous truncation studies indicated that the hydrophilic CT immediately flanking the transmembrane domain of a tail-anchored protein constitutes part of the sorting information but the CT tail itself could not direct fusion proteins to the chloroplast envelope (Lee et al., 2001, 2004; Dhanoa et al., 2010). On the other hand, the 56 residues of BsToc159 CT make up a hydrophilic tail, which could mediate the targeting of ca. 60% of EGFP to the chloroplast surface independently of the putative membrane anchor (Figure 3B; Lung and Chuong, 2012). While the ChloroP predictor suggested a 51-residue length of the TP-like sorting signal at the CT end of BsToc159 (Lung and Chuong, 2012), we observed that BsToc159-C56 outperformed BsToc159-C50 in the targeting of EGFP to the chloroplast envelope of *Arabidopsis* protoplasts (Figures 3A,B). The higher efficiency of targeting by the C56 construct could indicate that a longer sorting signal is required in the non-native context of a protein fusion. Similarly, it has been shown that a TP length of more than ca. 60 amino acids is required for efficient translocation of a passenger protein into chloroplasts (Bionda et al., 2010). It remains to be determined if the BsToc159 CT and typical chloroplast TPs employ similar targeting and translocation machineries. Previously, the TPs of 208 plastid proteins were grouped into seven subgroups with distinct sequence motifs by hierarchical clustering (Lee et al., 2008). The publicly available algorithm produced by Lee et al. (2008) did not identify any of the consensus motifs from the BsToc159 sequence (data not shown). The critical chloroplast-sorting motifs of the BsToc159 CT may be unraveled by the equivalent alanine substitution approach used by Lee et al. (2008) in the future. In conclusion, we have multiple lines of evidence to support the notion that Toc159 is not a tail-anchored protein but is targeted to the chloroplast surface via a novel pathway under the guidance of a non-canonical sorting signal at the CT.

### Toc159 is unconventionally anchored to the chloroplast outer membrane

In addition to shedding light on the nature of a novel chloroplast-sorting signal within the BsToc159 CT, the present study has provided the first insight into a long-standing question regarding how the Toc159 receptor is associated with the chloroplast outer membrane. At the time of its discovery, independent researchers consistently observed a 52-kDa protease-protected product of Toc159 (formerly known as OEP86) in pea and Arabidopsis after “shaving” the cytosolically exposed proteins/protein domains from the surface of isolated chloroplasts by treatment with thermolysin, an outer membrane-impermeable protease (Waegemann et al., 1992; Hirsch et al., 1994; Kessler et al., 1994; Bölter et al., 1998; Bauer et al., 2000; Chen et al., 2000). The M-domain has been defined based on this biochemical evidence, but it has never been clear how, exactly, the entire C-terminal 52-kDa portion of Toc159 is associated with the chloroplast envelope. While no study has yet addressed this issue, it is of our particular interest to examine the nature of the membrane-anchor of Toc159. Proteins traversing the envelope membranes of endosymbiotic organelles are structurally classified into two groups: α-helical transmembrane proteins and β-barrel proteins (Lee et al., 2013, 2014). Previous hydrophilicity analyses ruled out the possibility of Toc159 belonging to the former family due to the absence of a transmembrane α-helix (Kessler et al., 1994; Lung and Chuong, 2012). Although secondary structure prediction using the M-domain sequence of BsToc159 as query identified 16 consecutive β-strands in the central region (designated as the “M2 region” in this study; Figure 5B), they are too short (2–8 residues per strand; mean = 5.1 residue per strand) to represent the membrane-spanning regions (6–25 residues per strand) of a β-barrel protein (Taylor et al., 2006), which is in agreement with the negative result from the PROFtmb β-barrel predictor program (data not shown). Based on sequence analyses, we believe that the CTs of the Toc159 isoforms form a non-canonical anchor to the chloroplast outer membrane. The high homology of the central region of the AtToc159 M-domain (the M2 region) with the lipid-binding domain of LpxD, a left-handed β-helical protein, is consistent with our secondary structure prediction suggesting the presence of a short β-strand-rich region in the M-domain of Toc159, and also fosters the idea that Toc159 is anchored to the chloroplast outer membrane in a non-α-helical and non-β-barrel-dependent manner. Furthermore, the ratio of mean ellipticity at 208 nm and 220 nm of recombinant AtToc159 M-domain suggests the presence of associated forms of the protein (Hoang et al., 2013). The associations could be intramolecular interactions such as those that occur in the coiled-coil or β-coil structures, and/or intermolecular interactions between M-domain monomers. The precise nature of the associations cannot be elucidated from the current circular dichroism data; but the presence of associated forms is consistent with the predicted secondary structure elements of the M-domain (Figure 5). In the absence of additional structural data, it is premature to conclude that Toc159 adopts a lipophilic β-helix for associating with the chloroplast outer membrane. However, multiple lines of evidence support the notion that the M2 region of Toc159 constitutes a non-canonical membrane anchor: (i) a portion of the M2 sequence was sufficient to confer resistance of BsToc159-C100, but not BsToc159-C56, to alkaline extraction (Figure 4); (ii) the 180 residues from the CT end of PsToc159 also anchored a fusion protein to the chloroplast envelope with resistance to alkaline extraction (Muckel and Soll, 1996); (iii) truncation of BsToc159-C100 in blocks of 10 residues (i.e., C90, C80 and C70) progressively abolished the plastid-associated signals of EGFP fusion proteins (Figure 2); and (iv) the formation of irregular punctate structures of BsToc159-C70, C80 and C90 signals could be attributed to the disruption of an ordered structure essential for chloroplast association. In the future, a more in-depth structural analysis of the Toc159 M-domain will provide additional information about the nature of the unconventional membrane anchor, which will lead to insights into the function and mechanism of action of the Toc159 receptor. For instance, a number of reports have documented the partitioning of Toc159 between the cytosol and the chloroplast envelope, which suggests the possibility that Toc159 is a cycling receptor for preprotein recognition (Hiltbrunner et al., 2001; Bauer et al., 2002; Lung and Chuong, 2012). Thus, an unconventional membrane anchor (e.g., β-helix), in contrast with a transmembrane α-helix or a β-barrel, may account for the reversible association of Toc159 with the chloroplast outer membrane in support of the “cycling” hypothesis for Toc159-facilitated targeting of chloroplast preproteins (Hiltbrunner et al., 2001; Ivanova et al., 2004; Smith, 2006).

### Specificity for the targeting of Toc159 to the plastid envelope

Our discovery of a novel sorting signal at the Toc159 CT raised another interesting question regarding factors that interact with the sorting signal to mediate the specific targeting of Toc159 to the chloroplast envelope. In this study, we showed that the TP-like sorting signal at the CT of BsToc159 (i.e., BsToc159-C56) could guide EGFP to chloroplasts of *A. thaliana* mesophyll protoplasts (Figure 3B) but not to plastids of onion epidermal cells (Figure 2B). Similarly, it has been shown that some TPs guided protein import preferentially into one plastid type over others (Wan et al., 1996; Yan et al., 2006). Elkehal et al. (2012) demonstrated that the different composition of lipids in chloroplast membranes could influence the Toc-mediated binding and import of preproteins into outer envelope vesicles, and more recently, Kim et al. (2014) have shown that lipids of the outer membrane serve as the receptor for AKR2A. In addition to the effect on protein import, the lipids are known to be determinants of the topology, folding and integration of membrane proteins (Schleiff et al., 2001; Dowhan and Bogdanov, 2009). However, no significant difference has been found in the glycerolipid composition of envelope membranes from chloroplasts and non-green plastids (Douce and Joyard, 1979), and it has been shown previously that the lipid composition of plastids does not change during the greening of wheat leaves (Bahl et al., 1976). Thus, we hypothesize that the distinctive subcellular localization patterns of BsToc159-C56 proteins in onion epidermal cells and *Arabidopsis* mesophyll protoplasts is not likely attributed to the lipid composition of the plastid envelope membranes. Alternatively, it could also be explained by the possibility that the species-specific properties of the *Bienertia* CT may contribute to this differential subcellular localization. Any negative correlation between the targeting efficiency of BsToc159-C56 to protoeoliposomes and their lipid compositions would reinforce the idea that some unknown proteinaceous factors interact with the sorting signal of Toc159 and mediate a specific subcellular sorting pathway. Due to the resemblance of the CT sorting signal of Toc159 to a typical chloroplast preprotein TP (Lung and Chuong, 2012), it is plausible that the Toc machinery plays a similar role in the recognition of Toc159 CTs at the chloroplast surface. In fact, Wallas et al. (2003) reported that both binding and insertion of AtToc159 proteins into proteoliposomes required Toc34 and Toc75. In this regard, the fact that different Toc complexes are assembled in green and non-green cell types with dissimilar substrate specificities (Bauer et al., 2000; Ivanova et al., 2004; Kubis et al., 2004; Smith et al., 2004; Dutta et al., 2014) is consistent with our observation of differential targeting of BsToc159-C56 proteins to etioplasts and chloroplasts (Figures 2B, 3B). In addition to the Toc machinery at the chloroplast surface, the general import pathway of chloroplast preproteins involves chaperones, co-chaperones and other cytosolic factors (for review, see Lee et al., 2013). Although tail-anchored membrane proteins could be sorted efficiently to the chloroplast envelope with high fidelity in the absence of any cytosolic factor, the efficiency was higher with the supplementation of complete cytosol, Hsp70 or Hsp90 (Kriechbaumer and Abell, 2012). Recently, it has been demonstrated that AKR2A functions as a cytosolic mediator for targeting of outer envelope membrane proteins such as OEP7, Toc34, and OEP9 to the chloroplast (Bae et al., 2008; Dhanoa et al., 2010; Richardson et al., 2014). Although a number of other cytosolic receptors and chaperones for the targeting of chloroplast outer envelope proteins have also been identified (for review, see Lee et al., 2013), no cytosolic factors that specifically interact with the sorting signal of Toc159 have yet been reported. Given the observation that the CT sequences of Toc159 isoforms from *B. sinuspersici* outperformed that of *A. thaliana* in the sorting of fusion proteins to the plastid envelope (Figures 6, 7), a thorough interactome study of the Toc159 CT would not only reveal additional details about the chloroplast-sorting pathway of Toc159 but also further our understanding of the mechanism of selective protein import into dimorphic chloroplasts in the single-cell C_4_ system.

### Conflict of interest statement

The authors declare that the research was conducted in the absence of any commercial or financial relationships that could be construed as a potential conflict of interest.
