# Supplementary material for: The C-terminus of Bienertia sinuspersici Toc159 contains essential elements for its targeting and anchorage to the chloroplast outer membrane
Source: Front Plant Sci. 2014 Dec 23;5:722. doi: 10.3389/fpls.2014.00722 (PMC4274882; doi:10.3389/fpls.2014.00722)
Supplement: Supplementary file 1 [file Table1.DOCX]

| **Table S1.** List of primers used for the construction of EGFP fusion constructs. | | | |
| --- | --- | --- | --- |
| Construct Name | Primer |  | Orientation |
|  | Name | Sequence (5´ to 3´) |  |
| BsToc159-C50 | Toc159F33 | AAAACTCGAGCTGTTGCATTGAACAATAAG | Sense |
|  | Toc159R20 | CGCGGATCCTTAATAAATAGAGTAGCTTGGACTG | Anti-sense |
| BsToc159-C56 | Toc159F28 | AAAACTCGAGCTTCAAAGATGGCTCTTCGTG | Sense |
|  | Toc159R20 | CGCGGATCCTTAATAAATAGAGTAGCTTGGACTG | Anti-sense |
| BsToc159-C60 | Toc159F34 | AAAACTCGAGCTGTCGGAAGGAATTCAAAGATG | Sense |
|  | Toc159R20 | CGCGGATCCTTAATAAATAGAGTAGCTTGGACTG | Anti-sense |
| BsToc159-C70 | Toc159F35 | AAAACTCGAGCTATCGGAGGAAACCTTCAGTC | Sense |
|  | Toc159R20 | CGCGGATCCTTAATAAATAGAGTAGCTTGGACTG | Anti-sense |
| BsToc159-C80 | Toc159F36 | AAAACTCGAGCTTCTTTGATGAAATGGAGAGG | Sense |
|  | Toc159R20 | CGCGGATCCTTAATAAATAGAGTAGCTTGGACTG | Anti-sense |
| BsToc159-C90 | Toc159F37 | AAAACTCGAGCTGTTGGGCAGGAACAATCTAC | Sense |
|  | Toc159R20 | CGCGGATCCTTAATAAATAGAGTAGCTTGGACTG | Anti-sense |
| BsToc159-C100 | Toc159F38 | AAAACTCGAGCTGAAGTACGACTCAGAGAAGCCGAT | Sense |
|  | Toc159R20 | CGCGGATCCTTAATAAATAGAGTAGCTTGGACTG | Anti-sense |
| BsToc159-C100Δ56 | Toc159F38 | AAAACTCGAGCTGAAGTACGACTCAGAGAAGCCGAT | Sense |
|  | Toc159R31 | AAAAGGATCCTTAATTCCTTCCGACAGAAATCTG | Anti-sense |
| AtToc159-C101 | AtToc159F7 | AAAACTCGAGCTGAGGTCAGGCTTAGGGAAGC | Sense |
|  | AtToc159R6 | AAAAGGATCCTTAGTACATGCTGTACTTGTCGTTC | Anti-sense |
| AtToc132-C97 | AtToc132F3 | AAAACTCGAGCTGAAGCTCAGTTGAGAGATAAAGATTAT | Sense |
|  | AtToc132R2 | AAAAGGATCCTCATTGTCCATATTGCGTTTG | Anti-sense |
| BsToc132-C96 | Toc132F27 | AAAACTCGAGCTGAGGCCACATTTAGGGACA | Sense |
|  | Toc132-R13 | CGCGGATCCTCAATATCCAAGAGGATGCTCT | Anti-sense |
| AtToc159-C101/BsCT | AtToc159F7 | AAAACTCGAGCTGAGGTCAGGCTTAGGGAAGC | Sense |
|  | AtToc159R7 | AAAGGATCCTTAATAAATAGAGTAGCTTGGACTGACACCAGGCTTGAATTTCTGGTAGATGGACATGGCAATTGG | Anti-sense |
| AtToc132-C97-BsCT | AtToc132F3 | AAAACTCGAGCTGAAGCTCAGTTGAGAGATAAAGATTAT | Sense |
|  | AtToc132R3 | AAAGGATCCTCAATATCCAAGAGGATGCTCTTGACGCTGGCCAAGTAGCTTCTTGAAGAGAGGAACA | Anti-sense |
| BsToc159-C100/AtCT | Toc159F38 | AAAACTCGAGCTGAAGTACGACTCAGAGAAGCCGAT | Sense |
|  | Toc159R33 | AAAGGATCCTTAGTACATGCTGTACTTGTCGTTCGTCGCTTCGGGTCGAATGCTCTTATAGATAGAAAGAGCAATTGGAACTAG | Anti-sense |
| BsToc132-C96/AtCT | Toc132F27 | AAAACTCGAGCTGAGGCCACATTTAGGGACA | Sense |
|  | Toc132R25 | AAAGGATCCTCATTGTCCATATTGCGTTTGCGGGTAATAATAACTTATAATCTTTCTGAGCAGAGGAAGA | Anti-sense |
| Restriction sites used for subcloning are underlined. | | | |
