# Supplementary material for: The C-terminus of Bienertia sinuspersici Toc159 contains essential elements for its targeting and anchorage to the chloroplast outer membrane
Source: Front Plant Sci. 2014 Dec 23;5:722. doi: 10.3389/fpls.2014.00722 (PMC4274882; doi:10.3389/fpls.2014.00722)
Supplement: Supplementary file 2 [file Table2.DOCX]

**Table S2.** Solutions for deconvoluted CD spectra using the CDSSTER method for AtToc159M_His_ set of reference proteins.

Reference α1 α2 Segments per Avg α Length β1 β2 Segments per Avg β Length Unordered

Set 100 Residues per Segment 100 Residues per Segment

CDSSTRSet4 0.17 0.17 4.267 7.87 0.14 0.08 4.011 5.402 0.22

CDSSTRSet7 0.25 0.2 5.006 8.997 0.11 0.07 3.708 4.895 0.24
